# A real-world pharmacovigilance analysis of FDA adverse event reporting system database for upadacitinib

**DOI:** 10.3389/fphar.2023.1200254

**Published:** 2023-08-17

**Authors:** Yan Wu, Meihao Wei, Jing Zhang

**Affiliations:** Sir Run Run Shaw Hospital, School of Medicine, Zhejiang University, Hangzhou, China

**Keywords:** adverse event, FAERS database, upadacitinib, therapeutic drug monitoring, pharmacovigilance

## Abstract

**Objective:** To mine the adverse drug event (ADE) signals of upadacitinib based on the Food and Drug Administration (FDA) Adverse Event Reporting System (FAERS) database to provide a reference for the safe clinical use of the drug.

**Methods:** The ADE data for upadacitinib from Q1 2004 to Q1 2023 in the FAERS database were retrieved, and data mining was performed using the reporting odds ratio and proportional reporting ratio.

**Results:** A total of 21,213 ADE reports for the primary suspect drug upadacitinib were obtained, involving 444 ADEs. Patients aged ≥60 years (21.48%) and female (70.11%) patients were at a higher risk of ADEs with upadacitinib. After data cleaning, 182 ADE signals from 19 system organ classes (SOCs) were obtained. Six of these SOCs that occurred more frequently and were not mentioned in the drug labeling information included renal and urinary system (1.09%), reproductive and breast diseases (1.14%), ear and labyrinth disorders (0.57%), psychiatric disease (0.57%), blood and lymphatic system disorders (0.57%), and endocrine disorders (0.57%). The top ten most frequent ADE signals reported for upadacitinib were mainly related to: infections and infestations (7), investigations (2), and skin and subcutaneous tissue disorders (1). The top 10 ADEs in signal intensity ranking were lip neoplasm, ureteral neoplasm, eczema herpeticum, vulvar dysplasia, mediastinum neoplasm, eosinopenia, herpes zoster cutaneous disseminated, eye ulcer, acne cystic, and *Moraxella* infection. The top 10 high-frequency events leading to serious adverse events were urinary tract infection (2.74%), herpes zoster (1.63%), diverticulitis (1.19%), bronchitis (0.68%), nasopharyngitis (0.68%), localised infection (0.66%), nephrolithiasis (0.66%), pulmonary thrombosis (0.66%), blood cholesterol increased (0.55%), and *Pneumocystis jirovecii* pneumonia (0.53%).

**Conclusion:** Clinicians should be vigilant to upadacitinib-induced events in systems not covered in the drug labeling information and to new and highly signaled ADEs to ensure the safe and effective use of upadacitinib.

## 1 Introduction

Janus kinase (JAK) inhibitors are small-molecule compounds that can block the signal transduction of the JAK/STAT (signal transducers and activators of transcription) signaling pathway (Clark et al., 2014). JAK inhibitors block the synthesis and secretion of various inflammatory factors, thus exerting anti-inflammatory and immunomodulatory effects (Xin et al., 2020). This finding provides an opportunity for treating primary immune deficiency diseases, hereditary autoimmune diseases, auto-inflammatory diseases, and hematological and oncological disorders (McInnes and Gravallese, 2021). Currently, upadacitinib has received much attention as the world’s first highly selective JAK inhibitor. Upadacitinib was launched in the United States on August 16, 2019 and is approved by the Food and Drug Administration (FDA) for the treatment of rheumatoid arthritis (RA), psoriatic arthritis (PsA), ulcerative colitis, atopic dermatitis, ankylosing spondylitis, and non-radiographic axial spondyloarthritis. The drug entered the Chinese market on February 24, 2022.

In addition to affecting pathogenic pathways, the JAK/STAT pathway is critical for normal signal transduction in the body (Banerjee et al., 2017). Therefore, while inhibiting the JAK/STAT pathway may alleviate certain inflammatory symptoms, it is also likely to inhibit the normal transmission of essential cytokines in the body (Clarke et al., 2021). In particular, when JAK inhibitors are unable to selectively inhibit specific disease-related signaling pathways, they will inevitably have an impact on overall cytokine expression in the body. Non-selective JAK inhibitors, such as tofacitinib, have been found to have a high incidence of adverse events such as infections, cardiovascular disease, tumors, and liver injury in clinical trials (Ytterberg et al., 2022). Upadacitinib selectively inhibits the JAK1 pathway, and the compound is 60 and 100 times more selective for JAK1 over JAK2 and JAK3, respectively, at the cellular level (Parmentier et al., 2018). Disinhibition of JAK2 may lead to thrombocytopenia and anemia, and disinhibition of JAK3 may lead to a lack of T and B cell activity, which can lead to immunodeficiency and infections (Choy, 2019).

However, the adverse effects of upadacitinib should not be ignored. In a systematic review and network meta-analysis, Lasa et al. (2022) found that upadacitinib ranked highest in adverse effects compared to other biologics and small molecules used to treat patients with moderate-to-severe ulcerative colitis. In recent years, more attention has been paid to the adverse effects of JAK inhibitors in terms of thrombosis (Setyawan et al., 2021; Dong et al., 2022). There is, however, a lack of data on other side effects of upadacitinib. To this end, we study aimed to analyze the real-world safety of upadacitinib by mining the latest data in the FDA Adverse Event Reporting System (FAERS) database.

## 2 Materials and methods

### 2.1 Data source

The data used in this study were obtained from the FAERS database, which has been open to the public since 2004 and collects adverse drug events (ADEs) from the world. The data are spontaneously reported by medical professionals, patients, and pharmaceutical companies from different regions, and are extremely voluminous and not limited by space and time, allowing for the early detection of ADE signals and providing a basis for the safe clinical use of drugs (Zhai et al., 2019). In this study, the FAERS database was accessed through the OpenVigil 2.1 data platform. This platform is a pharmacovigilance tool validated by scholars such as Bohm, University of Kiel, Germany (Bohm et al., 2016). Due to spontaneous reporting, the FAERS database has flaws like inadequate reporting data, irregular reporting, and duplicate reporting. In contrast, this platform only loads reports with complete case information from the FAERS database and performs subsequent cleaning, so the total number of ADE frequencies may be slightly less than that of the FAERS database. However, the quality of data and results based on this platform analysis is more reliable due to the exclusion of incomplete reports. This study was conducted using the FAERS database. “Upadacitinib”, “Rinvoq”, and “ABT-494” were used as search terms, choosing “primary suspect” as the drug role, and the search period was from Q1 2004 to Q1 2023.

### 2.2 Data mining and cleaning

Frequency methods were used to detect ADE signals of upadacitinib, including the reporting odds ratio (ROR) and proportional reporting ratio (PRR) in the proportional imbalance method. In this method, the target ADE occurrence ratio of the target drug is compared with that of all other drugs. If the target ADE occurrence ratio is greater than the set threshold, it is considered as imbalance, which indicates the generation of potential ADE signal. Both methods are based on the disproportionality fourfold table (Supplementary Table S1). The ROR value, PRR value, and the corresponding 95% confidence interval (95% CI) were calculated according to the formulae (Sakaeda et al., 2013; Bohm et al., 2021). The formulae and thresholds for the ROR and PRR methods are shown in Supplementary Table S2. ADEs that met both the above ROR and PRR signal requirements were included and analyzed in this study. The internationally used methods for signal mining of ADEs are proportional imbalance analysis, including the PRR method, ROR method, Bayesian Confidence Propagation Neural Network (BCPNN), and Gamma Poisson Shrinker (GPS) (Kubota et al., 2004). The frequency method is simple to calculate and highly sensitive, but the possibility of false positives is high when the number of adverse events is very small; the Bayesian method is stable, but the calculation is complex and the signal detection time is relatively lagging. Therefore, both the PRR method and the ROR method were used in this study to improve the sensitivity and specificity of ADE signal detection. The higher the ROR and PRR, the stronger the ADE signal and the stronger the statistical relationship between the target drug and the target ADE (Sakaeda et al., 2013).

OpenVigil 2.1 data platform cleanses two files based on demographic information and drug information, retaining only those cases where all drugs in the report are accurately identified. After removing the duplicate individual safety reports, to reduce the “indication bias”, we screened excluded the indication-related signals and system organ classes (SOCs) not related to drug therapy, such as injury, poisoning, and procedural complications, product issues, surgical and medical procedures, and social circumstances.

### 2.3 Statistical analysis

The ADEs were categorized and described according to the preferred term (PT) and SOC in the International Medical Dictionary for Regulatory Activities (MedDRA), version 25.0 (Tieu and Breder, 2018). R language version 4.2.1 and Microsoft Excel 2019 were used to process the data.

## 3 Results

### 3.1 Descriptive results

After data cleaning, a total of 21213 adverse drug event (ADE) reports for upadacitinib were retrieved from Q1 2004 to Q1 2023, and 444 ADE signals were detected. In terms of gender composition, the number of females (14872 cases) was higher than that of males (5043 cases). Excluding 58.84% of patients of unknown age, fewer than 1% of the patients were under the age of 18 (0.42%), 19.26% were between the ages of 18 and 59, and 21.48% were over the age of 60. Reports originated from 51 countries, among which the top five countries were the United States, Canada, Japan, Germany, and Brazil, accounting for 79.72% of the total reports. Basic information regarding ADE reports concerning upadacitinib is presented in Table 1.

**TABLE 1:** Basic information of upadacitinib ADE reports.

	N. Of reported ADEs	Ratio (%)
Gender		
Male	5043	23.77
Female	14872	70.11
Unknown	1298	6.12
Age group, (y)		
<18	89	0.42
18–59	4085	19.26
≥60	4557	21.48
Unknown	12482	58.84
Reported Countries		
United States	15134	71.34
Canada	779	3.67
Japan	393	1.85
Germany	383	1.81
Brazil	222	1.05

### 3.2 ADE signals and organs involved in upadacitinib

Using the PRR and ROR methods, 444 ADE signals for upadacitinib were filtered according to the threshold value. After data cleaning, 182 signals remained, involving 19 SOCs, with a cumulative ADE frequency of 6100. The results showed that the SOCs with a high number of signals were infections and infestations (42.31%), neoplasms benign, malignant and unspecified (incl cysts and polyps) (9.89%), investigations (9.34%), and skin and subcutaneous tissue disorders (7.69%). Six of these SOCs identified were not mentioned in the drug labeling information for upadacitinib: renal and urinary system (1.09%), reproductive and breast diseases (1.14%), ear and labyrinth disorders (0.57%), psychiatric disease (0.57%), blood and lymphatic system disorders (0.57%), and endocrine disorders (0.57%).

### 3.3 ADE frequency of upadacitinib

Sorted by frequency, the top ten most frequent ADE signals reported for upadacitinib were mainly related to: infections and infestations (7), investigations (2), and skin and subcutaneous tissue disorders (1) as detailed in Table 2.

**TABLE 2:** Top 10 ADE frequency of upadacitinib

PT	SOC	Frequency	PRR (χ2)	ROR (95%CI)
Nasopharyngitis	Infections and infestations	465	2.946 (597.479)	2.99 (2.727–3.278)
Urinary tract infection	Infections and infestations	439	3.016 (590.619)	3.058 (2.782–3.362)
Acne	Skin and subcutaneous tissue disorders	407	12.867 (4356.46)	13.099 (11.863–14.464)
Infection	Infections and infestations	304	2.446 (258.89)	2.467 (2.202–2.763)
Herpes zoster	Infections and infestations	304	5.858 (1211.036)	5.928 (5.291–6.643)
Sinusitis	Infections and infestations	278	3.166 (409.242)	3.194 (2.837–3.597)
Blood cholesterol increased	Investigations	189	3.919 (406.277)	3.945 (3.417–4.555)
Bronchitis	Infections and infestations	144	2.123 (84.351)	2.13
				(1.808–2.51)
Hepatic enzyme increased	Investigations	137	2.323 (101.801)	2.332
				(1.97–2.759)
Upper respiratory tract infection	Infections and infestations	133	3.305 (210.802)	3.32
				(2.798–3.939)

### 3.4 Signal strength of ADEs of upadacitinib

The 182 upadacitinib ADE signals obtained were analyzed using the PRR method and the ROR method. The results of sorting by the PRR and the ROR methods are consistent. The top ten ADEs in the signal intensity ranking were all closely correlated with upadacitinib: lip neoplasm, ureteral neoplasm, eczema herpeticum, vulvar dysplasia, mediastinum neoplasm, eosinopenia, herpes zoster cutaneous disseminated, eye ulcer, acne cystic, and *Moraxella* infection, as shown in Table 3. Except for unknown age, 92.3% of all malignancies occurred in older patients (≥53 years).

**TABLE 3:** Top 10 signal strength of ADEs of upadacitinib

PT	SOC	N.	PRR (χ2)	ROR (95%CI)
Lip neoplasm	Neoplasms benign, malignant and unspecified (incl cysts and polyps)	3	47.631 (87.335)	47.637 (14.704–154.335)
Ureteral neoplasm	Neoplasms benign, malignant and unspecified (incl cysts and polyps)	3	47.631 (87.335)	47.637 (14.704–154.335)
Eczema herpeticum	Infections and infestations	16	46.188 (615.686)	46.222 (27.795–76.864)
Vulvar dysplasia	Reproductive system and breast disorders	4	36.565 (99.037)	36.572 (13.329–100.346)
Mediastinum neoplasm	Neoplasms benign, malignant and unspecified (incl cysts and polyps)	4	30.94 (83.555)	30.945 (11.328–84.532)
Eosinopenia	Blood and lymphatic system disorders	3	26.231 (47.657)	26.235 (8.257–83.356)
Herpes zoster cutaneous disseminated	Infections and infestations	4	20.985 (55.499)	20.989 (7.745–56.882)
Eye ulcer	Eye disorders	6	14.308 (60.112)	14.312 (6.369–32.162)
acne cystic	Skin and subcutaneous tissue disorders	19	13.696 (206.396)	13.707 (8.697–21.603)
*Moraxella* infection	Infections and infestations	3	13.116 (22.057)	13.117 (4.179–41.176)

### 3.5 Signals of serious adverse events with upadacitinib

After removing ADEs that did not specify the outcome of the adverse event, the clinical outcomes were analyzed and the frequency of serious adverse event (SAE) signals leading to death, life-threatening events, hospitalization or prolongation of the patient’s hospital stay, disability, and teratogenicity were collected and ranked. Overall, 22.2% of upadacitinib reports were associated with serious outcomes. The top ten most frequent occurrences were urinary tract infection (2.74%), herpes zoster (1.63%), diverticulitis (1.19%), bronchitis (0.68%), nasopharyngitis (0.68%), localised infection (0.66%), nephrolithiasis (0.66%), pulmonary thrombosis (0.66%), blood cholesterol increased (0.55%), and *Pneumocystis jirovecii* pneumonia (0.53%). Among them, nephrolithiasis (0.66%) was not mentioned in the drug labeling information.

## 4 Discussion

This study discovered that there were more ADE reports from female patients than from male patients for upadacitinib (14,872 and 5,043,respectively). Hunter T M et al. found a 1:4 ratio of the incidence of male-to-female patients with RA in the United States (Hunter et al., 2017), consistent with our findings with the ratio of ADE reports. In terms of age, the frequency of adverse reactions was higher in older individuals (≥60 years). Approximately 45% of the patients with RA are older than 65 years (Hunter et al., 2017), which is a patient group that may be associated with an increased risk of serious infections (Peng et al., 2020).

In this study, 182 signals involving 19 SOCs were mined. SOCs with a higher frequency of occurrence and more signals mainly focus on infections and infestations, investigations, neoplasms benign, malignant and unspecified (incl cysts and polyps), gastrointestinal disorders, vascular disorders, pneumonia, infection, herpes zoster, sinusitis, thrombosis, localised infection, and skin cancer. These ADEs are frequently reported, and are stated in the drug labeling information. Specific ADEs mentioned in the instructions, such as severe infection, tuberculosis, opportunistic infection, malignancy, gastrointestinal perforation, thrombosis, elevated hepatic transaminases, elevated lipids, and anemia were all detected in this study, which further verified the reliability of the current study.

It can be seen from the results of this study that most ADEs were concentrated in the infections and infestations SOC, both in terms of signal percentage (42.13%), frequency of occurrence (55.62%), and leading to SAEs, which is consistent with the results of previous safety trials of upadacitinib (Sandborn et al., 2020; McInnes et al., 2021; Reich et al., 2021). McInnes et al. (2021) found that the incidence of infection with upadacitinib was 39.4%–43.3%, and in this study, the real-world incidence of infection slightly higher than in clinical trials, which may be related to real-world patient diversity. Urinary tract infections, which were the second most frequently reported, have been reported in a previous phase III clinical trial report (Cohen et al., 2021). The sites of infection were not specified in the infections section in the drug labeling information.

In addition to common ADEs, data from this study uncovered renal and urinary system, reproductive and breast diseases, ear and labyrinth disorders, psychiatric disease, blood and lymphatic system disorders, and endocrine disorders. Such ADEs not mentioned in the drug labeling information, warranting further study to determine the causal relationship between ADEs and the drug. In the top 10 ranking of upadacitinib ADEs in terms of signal strength, both tumors and infections had strong signal intensities, suggesting a high correlation. Inhibition of the JAK/STAT pathway leads to loss of immune cell function, which induces malignancy. Except for unknown age, 92.3% of all malignancies occurred in older patients (≥53 years), consistent with clinical trial results (Fleischmann et al., 2022). Older patients need more attention for tumorigenesis. Among the top ten signals leading to serious adverse reactions, nephrolithiasis was not mentioned in the drug labeling information. Liang et al. (2019) found that some long-noncoding RNAs (lncRNA), microRNAs (miRNA), and messenger RNAs (mRNA) in the urine of patients with kidney stones were significantly different from those of normal subjects. These RNAs play a key role in the JAK/STAT pathway, which may be potentially related to kidney stones. As upadacitinib has only been on the market for a short time, no case reports or studies of these ADE-related adverse reactions exist, however, a total of 31 such ADE reports can be found in the FAERS database. We believe that the present study provides additional information for clinical practice and suggests that physicians should be highly vigilant to the possibility of such ADEs as early as possible.

This study had some limitations: 1) due to upadacitinib only being approved for use for a relatively short time and considering that the FAERS database is spontaneously presented, there may be problems of missing reports and under-reporting of ADEs, resulting in a bias in the results of signal analysis; 2) the FAERS database does not provide the baseline conditions of patients, in terms of preexisting conditions and liver and kidney function, so it is impossible to determine the influence of these factors on the occurrence of ADE; 3) OpenVigil 2.1 data platform does not grab the information about the reporter from the FAERS database, and our results would have been more complete if this part of the information had been made available; 4) the ROR and PRR methods can only indicate the existence of a statistical correlation between the target drug and the target ADE and cannot indicate the causal relationship between them. The ADE signals that differ from the drug labeling information obtained in this study need to be further explored by reviewing new clinical data and research methods.

## 5 Conclusion

In conclusion, this study used the OpenVigil 2.1 data platform based on the FAERS database to mine the ADE signals of upadacitinib, eliminate incomplete information, and make the data analysis completer and more reliable, which can provide a reference for the safe use of upadacitinib in patients. Clinicians should be vigilant to the possibility of new ADEs identified in this study that are not detailed in the drug labeling information. Safety monitoring should be reinforced to effectively reduce the incidence of upadacitinib-related ADEs.

## Data Availability

The original contributions presented in the study are included in the article/Supplementary Material, further inquiries can be directed to the corresponding author.
