# Supplementary material for: A real-world pharmacovigilance analysis of FDA adverse event reporting system database for upadacitinib
Source: Front Pharmacol. 2023 Aug 17;14:1200254. doi: 10.3389/fphar.2023.1200254 (PMC10469920; doi:10.3389/fphar.2023.1200254)
Supplement: Supplementary file 1 [file Table1.DOCX]

**Table S1.** Two-by-two contingency table for disproportionality analyses

| **Item** | **Reports with the target AEs** | **All other AEs** | **Total** |
| --- | --- | --- | --- |
| **Reports with the target drug** | $\mathbf{a}$ | $\mathbf{b}$ | $\mathbf{a}$**+**$\mathbf{b}$ |
| **All other drugs** | $\mathbf{c}$ | $\mathbf{d}$ | $\mathbf{c}$**+**$\mathbf{d}$ |
| **Total** | $\mathbf{a}$**+**$\mathbf{c}$ | $\mathbf{b}$**+**$\mathbf{d}$ | $\mathbf{a}$**+**$\mathbf{b}$**+**$\mathbf{c}$**+**$\mathbf{d}$ |
