# Supplementary material for: A real-world pharmacovigilance analysis of FDA adverse event reporting system database for upadacitinib
Source: Front Pharmacol. 2023 Aug 17;14:1200254. doi: 10.3389/fphar.2023.1200254 (PMC10469920; doi:10.3389/fphar.2023.1200254)
Supplement: Supplementary file 2 [file Table2.DOCX]

**Table S2.** Summary of major algorithms used for signal detection

| **Algorithms** | **Equation** | **Criteria** |
| --- | --- | --- |
| **ROR** | **ROR=(**$\mathbf{a}$**/**$\mathbf{c}$**)/(**$\mathbf{b}$**/**$\mathbf{d}$**)=**$\mathbf{ad}$**/**$\mathbf{bc}$ | $\mathbf{a}$**≥3, 95%CI ＞1** |
|  | **SE(lnROR)=**$\sqrt{\mathbf{(}\frac{\mathbf{1}}{\mathbf{a}}\mathbf{+}\frac{\mathbf{1}}{\mathbf{b}}\mathbf{+}\frac{\mathbf{1}}{\mathbf{c}}\mathbf{+}\frac{\mathbf{1}}{\mathbf{d}}\mathbf{)}}$ |  |
|  | **95%CI=**$\mathbf{e}^{\boldsymbol{lnROR\pm1.96}\sqrt{\mathbf{(}\frac{\mathbf{1}}{\mathbf{a}}\mathbf{+}\frac{\mathbf{1}}{\mathbf{b}}\mathbf{+}\frac{\mathbf{1}}{\mathbf{c}}\mathbf{+}\frac{\mathbf{1}}{\mathbf{d}}\mathbf{)}}}$ |  |
| **PRR** | **PRR=**$\left[ \mathbf{a/(a+b)} \right]$**/**$\left[ \mathbf{c/(c+d)} \right]$ | $\mathbf{a}$**≥3,PRR≥2,χ2≥4** |
|  | **χ2=**$\left( \mathbf{ad-bc} \right)^{\mathbf{2}}\left( \mathbf{a+b+c+d} \right)\mathbf{/}\left[ \mathbf{(a+b)(c+d)(a+c)(b+d)} \right]$ |  |
